# Human cytomegalovirus haplotype reconstruction reveals high diversity due to superinfection and evidence of within-host recombination

**DOI:** 10.1073/pnas.1818130116

**Published:** 2019-02-28

**Authors:** Juliana Cudini, Sunando Roy, Charlotte J. Houldcroft, Josephine M. Bryant, Daniel P. Depledge, Helena Tutill, Paul Veys, Rachel Williams, Austen J. J. Worth, Asif U. Tamuri, Richard A. Goldstein, Judith Breuer

**Affiliations:** ^a^Division of Infection and Immunity, University College London, London WC1E 6BT, United Kingdom;; ^b^Infection, Immunity, Inflammation and Physiological Medicine, Institute of Child Health, University College London, London WC1N 1EH, United Kingdom;; ^c^Great Ormond Street Hospital for Children, London WC1N 3JH, United Kingdom;; ^d^Research Computing, University College London, London WC1E 6BT, United Kingdom

**Keywords:** human cytomegalovirus, diversity, whole genome sequencing, superinfection, recombination

## Abstract

Recent sequencing efforts have led to estimates of human cytomegalovirus (HCMV) genome-wide intrahost diversity that rival those of persistent RNA viruses [Renzette N, Bhattacharjee B, Jensen JD, Gibson L, Kowalik TF (2011) *PLoS Pathog* 7:e1001344]. Here, we deep sequence HCMV genomes recovered from single and longitudinally collected blood samples from immunocompromised children to show that the observations of high within-host HCMV nucleotide diversity are explained by the frequent occurrence of mixed infections caused by genetically distant strains. To confirm this finding, we reconstructed within-host viral haplotypes from short-read sequence data. We verify that within-host HCMV nucleotide diversity in unmixed infections is no greater than that of other DNA viruses analyzed by the same sequencing and bioinformatic methods and considerably less than that of human immunodeficiency and hepatitis C viruses. By resolving individual viral haplotypes within patients, we reconstruct the timing, likely origins, and natural history of superinfecting strains. We uncover evidence for within-host recombination between genetically distinct HCMV strains, observing the loss of the parental virus containing the nonrecombinant fragment. The data suggest selection for strains containing the recombinant fragment, generating testable hypotheses about HCMV evolution and pathogenesis. These results highlight that high HCMV diversity present in some samples is caused by coinfection with multiple distinct strains and provide reassurance that within the host diversity for single-strain HCMV infections is no greater than for other herpesviruses.

## Significance

Human cytomegalovirus (HCMV) is a major global cause of congenital disability and transplant-related morbidity. Excessive levels of within-host HCMV nucleotide diversity are attributed to unexpectedly high mutation rates. Here, we show that high HCMV diversity is due to the frequent presence of mixed infections with genetically distinct strains, whereas HCMV in nonmixed infections is no more diverse than other DNA viruses. Using serial patient samples, we reconstruct viral strain haplotypes to pinpoint the timing of HCMV superinfections occurring within the study sampling time frame and uncover within-host viral recombination. From these results, we identify likely sources of infection and demonstrate probable selection for recombinant viruses. These results generate new, yet testable, insights into putative viral and host drivers of HCMV evolution and pathogenesis.

HCMV, a geographically ubiquitous human-restricted herpesvirus, infects 45–100% of the adult population, depending on age and geographical region (1). Although generally asymptomatic, HCMV is a significant cause of morbidity and mortality in infants and patients with primary or acquired immunodeficiency with the potential to cause devastating and life-threatening symptoms including congenital defects, blindness, and organ transplant failure (1).

Although vaccines are under development, a potential hindrance to their efficacy lies in the substantial genetic variability observed across HCMV genomes, higher than that of any other human herpesvirus studied. Recent sequencing efforts have also revealed this heterogeneity may manifest within a single patient, leading to estimates of genome-wide intrahost diversity that rival those of persistent RNA viruses, such as HIV and hepatitis C virus (HCV) (2–6). Somewhat counterintuitively, the elevated diversity has been attributed to high levels of within-host de novo mutation (7) despite the low error rates of DNA polymerases (8). More recently, HCMV’s rapid rate of recombination (9) together with the high prevalence of coinfection with different HCMVs (5) have been mooted as a more plausible mechanism for variation (2, 3). Distinguishing diversity that is due to superinfections and/or recombination from diversity due to de novo mutation is critical to understanding intrahost HCMV evolution, particularly in relation to HCMV compartmentalization and how this impacts on congenital and transplant (5) infections as well as for the design of effective preventative and treatment measures.

Here, we make use of next generation deep sequencing data derived using the same standardized platforms to compare the within-host viral diversity of HCMV with nine other DNA and RNA viral species. Through longitudinal sampling of chronically HCMV-infected individuals and reconstruction of individual viral haplotypes, we hypothesize that previous estimates of HCMV diversity have been influenced by the presence of multiple strains in a single sample. We find infections with single strains of HCMV to be no more diverse within a host than other human herpesviruses and considerably less variable than chronic RNA viruses, such as HIV and HCV. By tracking changes in viral population structure over time, we gain insight into superinfection events, their likely timing, and observe how recombination in the face of selective forces, such as drug resistance contribute to HCMV intrahost evolution.

## Results

### Within-Host Diversity of HCMV Segregates into “Low” and “High” Populations.

**Fig. 1.**
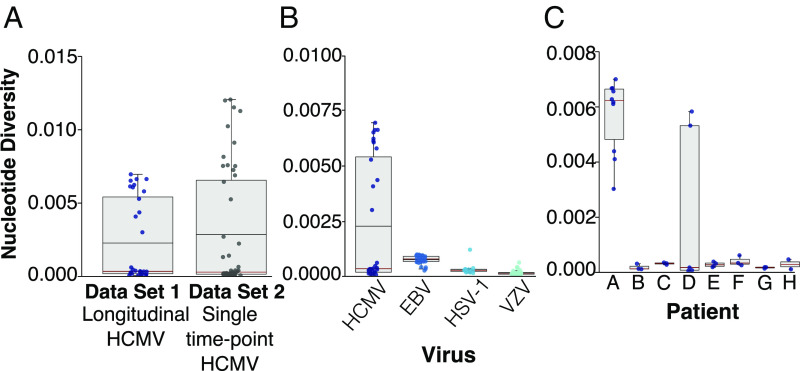
Box and whisker plot showing HCMV genome-wide within-host nucleotide diversity is dichotomous. (*A*) Genome-wide intrahost nucleotide diversity in longitudinal versus single time point HCMV samples. The circles represent individual samples. The mean values shown by the horizonal black line, and the median values shown by the red horizontal line. The box represents a 25–75% interquartile range. The maximum and minimum values are represented by vertical lines. (*B*) Comparison of HCMV diversity to other herpesviruses. (*C*) Nucleotide diversity within eight individual patients that make up the HCMV longitudinal dataset 1.

Our primary HCMV datasets consisted of 34 samples collected from eight immune-compromised children over the course of their infections (set A) as described in Houldcroft et al. (10) (*SI Appendix*, Table S1). Genome-wide within-host nucleotide diversity (π) was calculated for each sample (*SI Appendix*, *Methods*). We found that, on average, observed within-host HCMV diversity for set A (Fig. 1*A*) was higher than that of other herpesviruses [EBV, HSV, and varicella zoster virus (VZV)] (Fig. 1*B*) but similar to the diversity seen in 50 samples (set B) collected at a single time point from both 35 immunosuppressed children and adults (transplant recipients, HIV positive or with primary immunodeficiencies) and 15 congenitally infected patients (Fig. 1*A*). In both sets, HCMV sample diversity separated into higher and lower groups with the majority demonstrating comparatively low within-host diversity (Fig. 1*A*). Separating the samples in set A by patient revealed that the mean diversity value obtained for the group of eight patients was inflated by the outlying “high diversity” samples taken from patients A and D (Fig. 1*C*). Across all HCMV sample sets A and B), median genome-wide within-host diversity was calculated to be 0.000306 (9), similar to that observed for other herpesviruses, as well as previous estimates of HCMV diversity.

### Evidence for Mixed HCMV Infections.

**Fig. 2.**
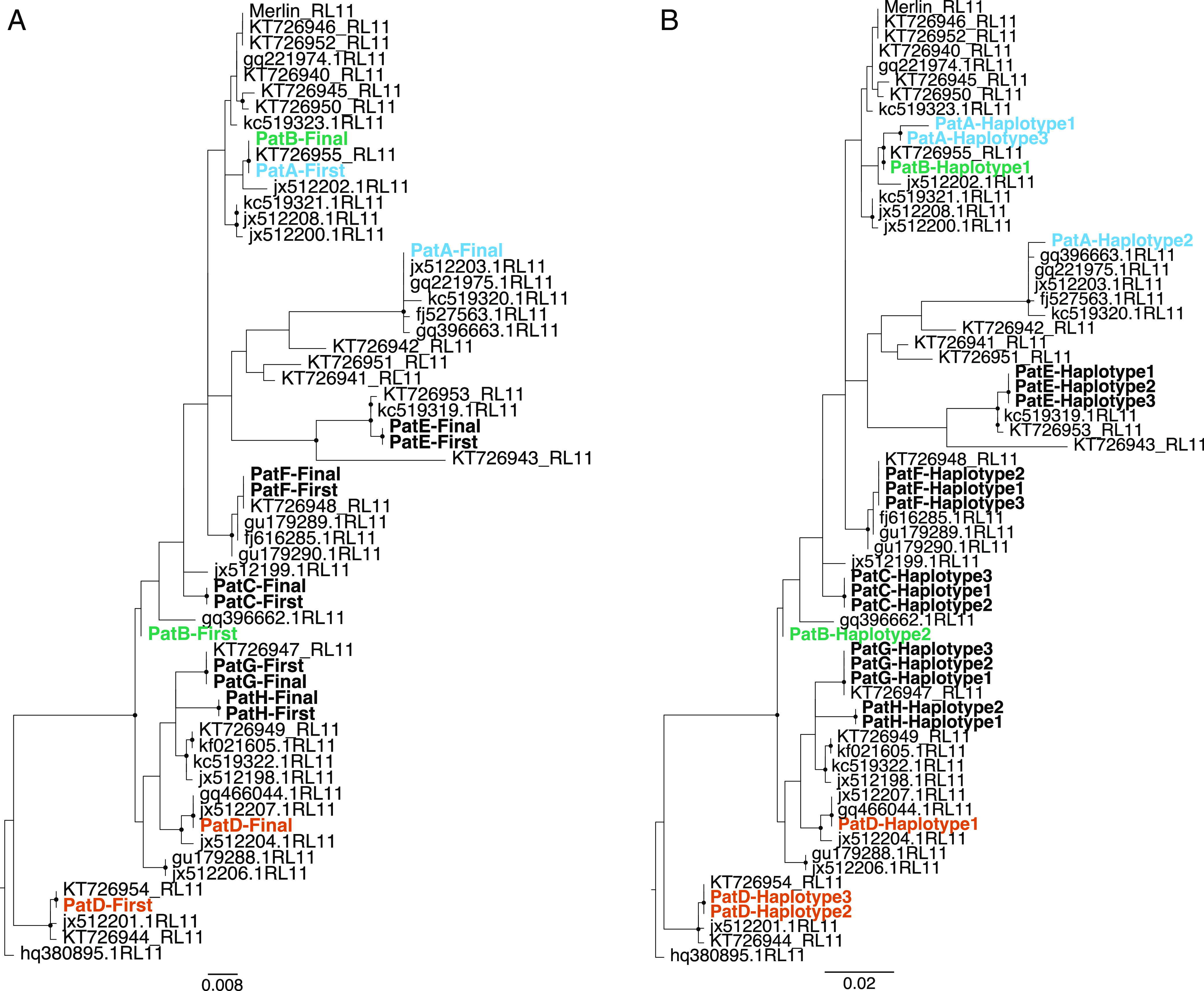
Multiple HCMV infections are present in high-diversity samples. Maximum likelihood phylogenies analysis of the RL11 region from HCMV sequences. (*A*) Consensus HCMV sequences taken from the first and last sampling time points from each of the eight patients in the longitudinally samples cohort. (*B*) Haplotype sequences reconstructed using longitudinal HCMV samples taken from each patient. GenBank sequences are in gray. Sequences from the same patients that cluster paraphyletically (A, B, and D) are colored by patient. Patient sequences that cluster monophyletically are in bold black. Topological robustness was assessed using 1,000 bootstrap replicates. The nodes with black circles represent those with bootstrap scores of >70.

To determine if the high diversity seen in HCMV sequences from patients A and D was the result of mixed infection or, instead, arose from evolution within the patient over time, we reconstructed a phylogenetic tree containing these samples together with highly divergent HCMV strains downloaded from GenBank (Fig. 2). We and others have previously shown that robust whole-genome phylogenies are difficult to obtain for HCMV given its high propensity for recombination (9, 11). To circumvent this problem, we chose the 10 kb RL11D region for our analyses as it had a higher level of linkage disequilibrium, indicating less recombination, as well as sufficient diversity to resolve distinct strains (5, 9, 11). We found that, whereas sequences from the first and final time points from most patients were sister taxa, sequences from patients A and D were paraphyletic, clustering with high confidence into separate clades (Fig. 2*A*). Patient B also showed separation between the first and the last sequences despite low diversity (Fig. 1*C*). The separation of these sequences into distinct clades indicates that the strains present in the final sample from patients A, B, and D are distinct from those present in the first sample and arose independently as a result of an additional HCMV infection rather than evolving from the initial strain within the patient.

To confirm the presence of mixed strains in samples from patients A, B, and D, we reconstructed whole-genome HCMV haplotypes from longitudinal sequence data using the newly developed program *HaROLD* (haplotype reconstruction of longitudinal deep-sequencing data) (12). The term haplotype in this context is used to describe a distinct collection of alleles present on the same viral genome, arising from a novel mutation on a preexisting backbone or a phylogenetically distinct cocirculating viral strain. The number of haplotypes was determined by maximizing the logarithmic likelihood, resulting in three haplotypes for patients A, C, D, E, F, and G and two each for patients B and H (*SI Appendix*, Fig. S1). From these reconstructed sequences, we generated a second RL11 phylogeny. Haplotype sequences from patients A (A1 and A3), B (B1 and B2), and D (D1 and D3) clustered with high confidence into separate clades, corroborating the original results (Fig. 2*B*). For all other samples, within-patient RL11 patient haplotypes clustered along the same branch as did haplotypes A1 and A2 from patient A and D1 and D2 from patient D, suggesting common ancestry or potential within-host evolution (Fig. 2). To ensure that these findings were not confined to the RL11 gene complex, we calculated the maximum pairwise differences between predicted HCMV whole genome haplotypes for patients A (A1 and A3), B (B1 and B2), and D (D1 and D3) to be equal to those between unrelated sequences obtained from GenBank and higher than for predicted haplotypes in the other patients (*SI Appendix*, Fig. S2).

### Measures of Within-Host Diversity Increase as a Function of Population Mixture.

**Fig. 3.**
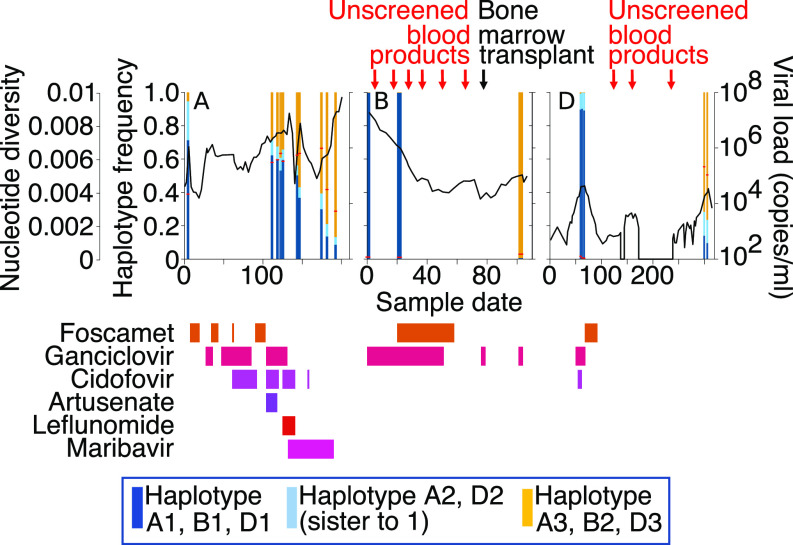
Haplotype abundances over time for patients A, B, and D with mixed (paraphyletic) infections. Haplotype frequencies in each sample are shown. The dark and light blue haplotypes are sister taxa, whereas the yellow haplotypes are paraphyletic in Fig. 2. The viral load is represented by the black line, and sample diversity is represented by a horizonal red bar. Antiviral drugs prescribed and their timings are shown in shades of pink and red. The timing of HSCT and blood products administered to patients B and D are shown.

To understand the timing and course of superinfections occurring within Patients A, B, and D, we calculated the abundance of each reconstructed haplotype sequence over time and related this to sample diversity (Fig. 3). All Patient B samples and the first three from Patient D showed low diversity, reflecting the predominance of one haplotype in each sample at nearly 100%. Haplotypes present at frequencies below 1% (0.1–0.8%) in patients B and D, corresponding to ∼1 read per site, were considered indistinguishable from noise and were ignored. On this basis, we conclude that Patient B was initially infected with a single virus B1 and Patient D with two closely related haplotypes; D2 having evolved from D1. In both cases, superinfection with a second virus could have occurred. In Patient B, virus B2 had completely replaced virus B1 by the time of sampling at day 105, maintaining the low nucleotide diversity (Fig. 3). Although Patient B had received a stem cell transplant at day 80, this was HCMV negative and, therefore, not the source of virus B2. Instead, HCMV-unscreened blood products administered before the transplant, in line with clinical policy for transplanted patients who are themselves already HCMV positive, are a more likely source (Fig. 3). In Patient D, haplotypes D1 and D2 disappeared, being undetectable by day 175, some 5 mo following a stem cell transplant (Fig. 3). HCMV viremia was again detected on day 205 with the new haplotype D3 being the most abundant by the time of sampling at day 300. Again, HCMV-unscreened blood products may have been the source of virus D3.

In contrast, by the time of the first sample analyzed, Patient A was already infected with the closely related haplotypes A1 and A2 (95%) and the paraphyletic virus A3 (5%) with A3 replacing A1 to rise to near fixation by day 190 (Fig. 3). For patients A and D, diversity was higher across time points where paraphyletic haplotypes (A1 and A2 versus A3 and D1 and D2 versus D3) were present at frequencies of 10% or more being highest when these mixtures approached 50:50 (Fig. 3). To confirm that nucleotide diversity is associated with the presence of multiple genetically distinct HCMV strains present in the same sample, we remapped reads for patients A and D using the reconstructed haplotype sequences as a reference. Strikingly, we find that, for both patients A and D, estimates of nucleotide diversity for individual haplotypes was similar to that observed for singly infected patients (*SI Appendix*, Fig. S3).

### Haplotype Reconstruction Across the Genome.

**Fig. 4.**
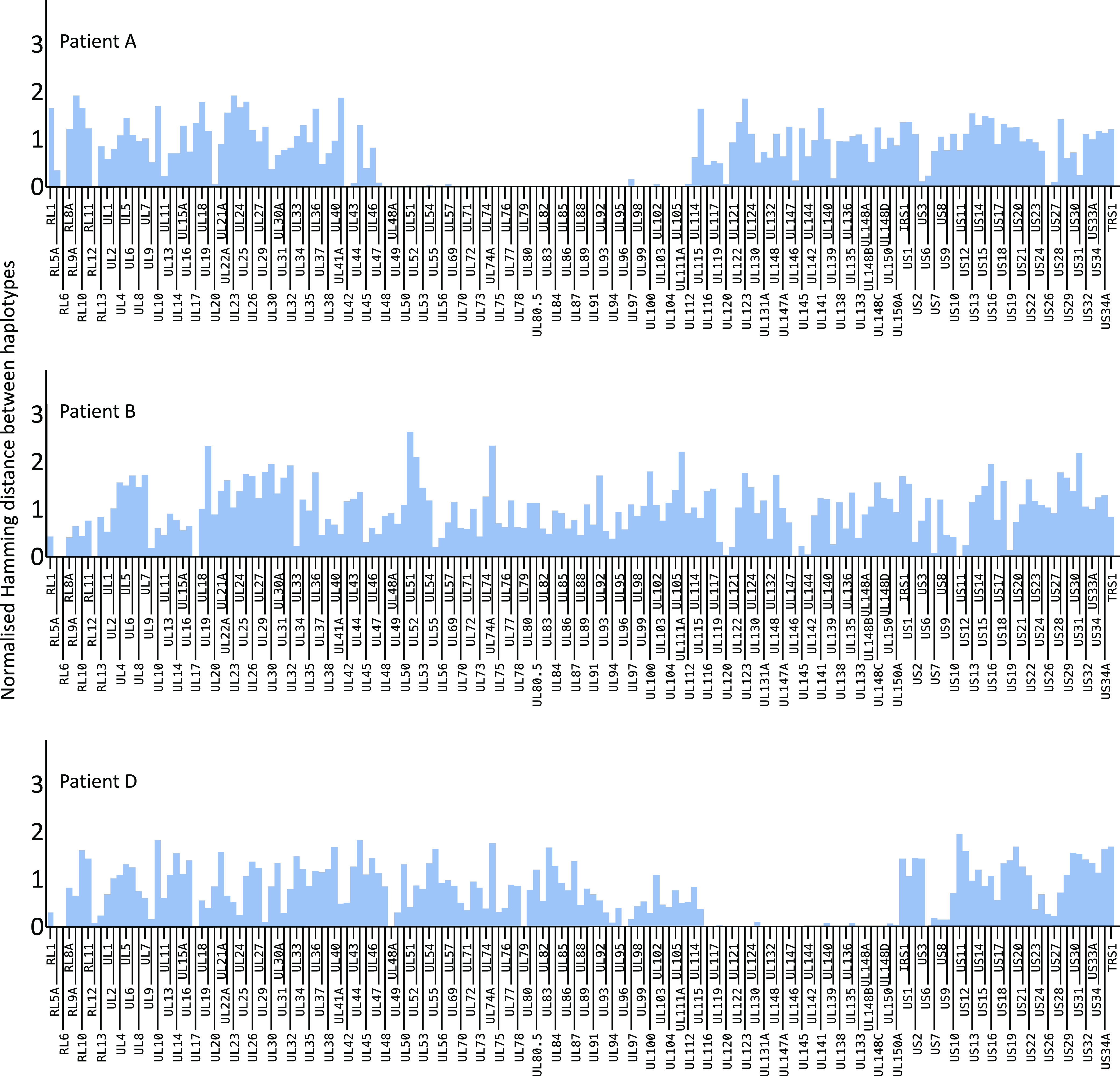
Genome-wide pairwise genetic distance among paraphyletic haplotypes in patients A, B, and D. The blue bars represent genetic distance among ORFs for paraphyletic haplotypes A1 and A3, B1 and B2, and D1 and D3, normalized for average pairwise distance between unrelated GenBank HCMV sequences (red line).

To examine genome-wide genetic distance among haplotypes in mixed infections, we calculated pairwise distances between haplotypes for the different ORFs. The values obtained were normalized by dividing by the average pairwise distance observed in the same ORF between unrelated GenBank sequences. We analyzed this distance in Patients A and D, both of whom had evidence for persisting HCMV coinfection with distinct haplotypes (Fig. 3). Also, for Patient B, in whom infection with distinct haplotypes occurred without evidence of coinfection in the samples tested and Patients C, E, F, and G for whom there was no evidence of multiple infection (Fig. 4 and *SI Appendix*, Fig. S4). For patient H, haplotype H1 was present at low levels (≤5%) in both samples sequenced with average read depths of <10 making gene-by-gene sequence comparisons unreliable. The median relative sequence difference between the two HCMV haplotypes infecting patient B (B1 and B2) is approximately equal to that of unrelated GenBank sequences across the whole genome (Fig. 4). For Patients A and D, the genetic distances between the phylogenetically distant haplotypes (A1 versus A3 and D1 versus D3) was also like that seen between unrelated GenBank sequences, apart from one region in both cases where there was apparent sequence identity between otherwise distinct haplotypes (Fig. 4). Sequences for haplotypes A1 and A3 were almost identical between nucleotide positions from 60 and 160 kb, i.e., ORFs UL48–UL112, and a similar finding was observed for haplotypes D1 and D3 between 170 and 190 kb (ORFs UL115–UL150A). These results are most parsimoniously explained by recombination. For patient D, virus D3 must have recombined with viruses D1/D2 with the loss of the parental D3 UL115–UL150A region. In the case of patient A where all three viruses were present in the first sample sequenced, the recombinant UL48–UL112 sequence was present in the highly abundant A1 and A2, but there were too few reads to determine accurately the sequence of this region in A3. By sample 2, all three A haplotypes were confirmed to be identical in this region within which lies the DNA polymerase (UL54) and protein kinase (UL97) genes both of which are targets for antivirals used to treat Patient A (Fig. 3 and *SI Appendix*, Fig. S5). Plotting the frequencies of mutations known to confer resistance to the drugs administered as well as substitutions not known to confer resistance but occurring at resistance loci (*SI Appendix*, Fig. S5), provided insight into the putative role of drug resistance in driving haplotype frequencies and recombination (*SI Appendix*, Fig. S5). Haplotypes in Patients C, E, F, and G, which clustered monophyletically within their respective hosts, were largely identical across their genomes confirming within host evolution (*SI Appendix*, Fig. S4).

### HCMV Within-Host Diversity Is Less than That of Chronic RNA Viruses.

**Fig. 5.**
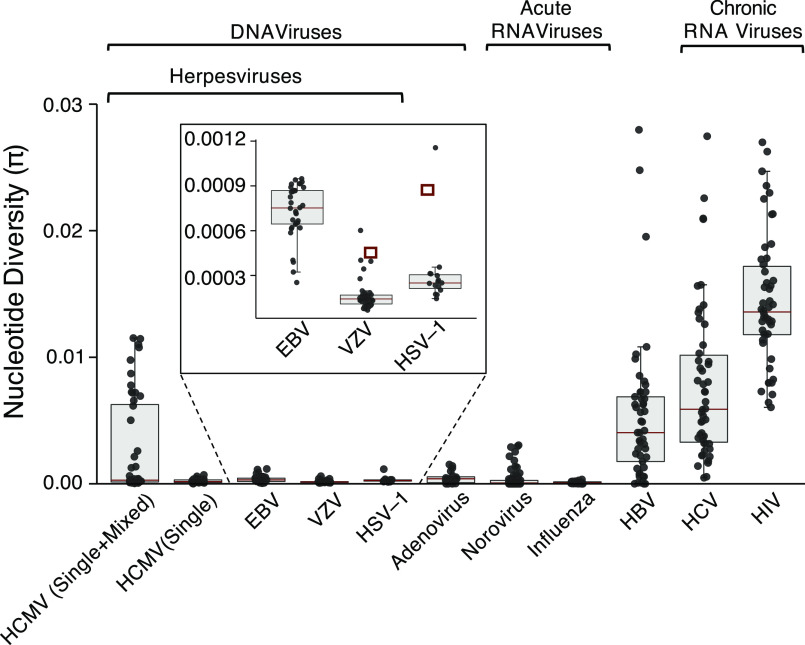
Genome-wide within-host nucleotide diversity across DNA and RNA viruses. The circles represent individual samples. Mean diversity represented by a black horizontal line. Median diversity represented by a red horizontal line. The box represents a 25–75% interquartile range. The maximum and minimum values are represented by vertical lines. The number of samples in each dataset shown above the plot. The “HCMV (Single+Mixed)” label represents diversity calculated from all samples mapped to reference, whereas the “HCMV (Single)” label represents all samples where reads from patients with mixed infections were remapped to resolve single-infection diversity. The circled points represent known mixed infections.

Having mapped read data back to haplotypes and corrected for the presence of mixed infections (*SI Appendix*, Fig. S3), we calculated the average genome-wide HCMV within-host nucleotide diversity to be 8.35 × 10^−5^, which is similar to the mean values observed for other DNA viruses (Figs. 1 and 5). We found the most outlying samples for VZV and HSV-1 (Fig. 5, *Inset*) to contain mixed strain infections, whereas none of the EBV samples in this study were mixed. Notably, for HSV-1 and VZV, the mixed outlier samples did not differ from the mean as much as HCMV, likely due to the lower population-level variability between strains of these viruses (11). Compared with RNA viral genomes that had been sequenced and analyzed in the same pipeline, within-host HCMV diversity was found to be considerably less than even the least diverse of canonical “hyperdiverse” viruses including hepatitis B, C, and HIV, with HIV exhibiting the highest average within-host diversity of any of the viruses tested (Fig. 5).

## Discussion

By reconstructing haplotypes from short read sequenced data, we demonstrate that the most parsimonious explanation for observed high within-host HCMV diversity is the frequent presence of mixed infections (Figs. 2 and 3). Making use of a unique collection of DNA and RNA viruses for which sequencing, quality control, variant calling, and calculation of nucleotide diversity had been standardized, we overcome the methodological biases that can accompany between-study comparisons (13) to confirm that within-host diversity of monophyletic HCMV infections is similar to that seen for other DNA viruses and significantly lower than the mean diversity of persistent RNA viral infections (Figs. 1 and 5).

Our findings provide some reassurance for the development of vaccines against HCMV as unlike for hyperdiverse RNA viruses, vaccine-escape mutations are not likely to occur. Notwithstanding, with mixed infections being common and HCMV having much greater diversity at the population level than that seen for other herpesviruses including HSV, VZV, or EBV (11), there remain significant challenges for the design of effective vaccines to prevent HCMV infection (14).

The frequency of mixed infection in HCMV infections can be as high as 61% (15–17), particularly, in the immunocompromised (5, 9, 11) and mixed HCMV infections has been associated with poorer clinical outcomes (10, 18, 19). Viral recombination, a natural corollary of mixed infection, is thought to be a major influence on HCMV pathogenesis, for example, in generating new glycoprotein B genotypes (20) [promoting viral entry to the cell (21)]. Mechanistic insights into this process, however, have been constrained by difficulties in deciphering the evolutionary dynamics of individual viruses within mixed infections. Sequencing methods that generate haplotype information from long reads are currently too error prone to give accurate outputs or requiring of too much viral DNA to work directly on clinical material. Existing computational methods used for pathogen haplotype reconstruction are largely designed for highly variable RNA viruses (22, 23) or, based on cluster analysis and read phasing, exclude too much genetic information for robust phylogenetic-based analysis (24). Instead, we used a likelihood-based model to resolve genetically divergent HCMV haplotypes from short read data and applied it to patients (A, B, and D) with multiple HCMV infections (12). Haplotypes for both Patient A and Patient D show long genome-wide genetic distances and distinct phylogenetic histories that suggest separate introductions of separate HCMV viruses rather than the evolution of one haplotype from the other (Fig. 2). Patient B showed complete haplotype (strain) replacement with no evidence of mixed infection in the samples tested. In contrast, although more than one HCMV haplotype were supported within Patient C, E, F, G, and H samples which have low within-host diversity, these are genetically similar (*SI Appendix*, Figs. S2 and S3), cluster monophyletically within a phylogenetic tree (Fig. 2) and are most parsimoniously explained by within-host evolution of a single strain.

Reconstructing haplotypes from short read data allowed us to calculate haplotype abundance over time to pinpoint the possibility that superinfection had occurred and its likely timing and to speculate on the origins of superinfecting strains. In Patients B and D, the superinfection could have originated from HCMV-unscreened blood transfusions that have been described, albeit rarely, to transmit HCMV (25, 26) (Fig. 3). Patient D achieved control of his initial HCMV infection approximately 5 mo following stem cell transplant with viruses D1 and D2 becoming undetectable by day 175. It is possible that the superinfection with D3 occurred between day 175 and day 300, the date of the next sample sequenced at a time when immunosuppression was minimal and T cell recovery well documented. All three haplotypes, D1, D2, and D3 were present at day 300, raising the possibility that superinfection with virus D3 triggered the reactivation of viruses D1 and D2 notwithstanding good immune function. Alternatively, D3 might have been present at undetectable levels in early samples, becoming detectable only in later samples. Cocirculation of all three viruses would have promoted the recombination observed among D3 and D1/D2. Since ORFs UL128–UL131 within the recombinant fragment, the code for the pentameric complex, which is required for endo- and epithelial cell tropisms (27) and UL132–UL150, part of the ULb′ region that is lost in fibroblast culture (28), contains natural killer immune evasion genes and chemokine paralogs (UL146/UL147) (29), acquisition of these genes may have altered D3 infectivity, evasion of innate immunity, and tropism. At the same time, retention of epitopes in proteins outside the recombinant region would have allowed D3 to evade the adaptive host immunity that controlled viruses D1 and D2. In the case of Patient A, haplotype reconstruction provided detailed insight that allowed us to speculate how recombination, mutation, and drug selection are likely to have driven the emergence of the highly resistant A3 virus, which outcompeted viruses A1 and A2 (*SI Appendix*, Fig. S5). The results have identified putative drug resistance mutations and provided tools to undertake more detailed analysis of HCMV control at the molecular level.

To summarize, we show no evidence for high HCMV mutation rates driving large HCMV diversity over the short evolutionary timescale. Instead, we confirm that mixed infection with distinct viral haplotypes occurs frequently and is the explanation for the observed high levels of within-patient HCMV diversity. By resolving individual viral haplotypes within patients, we can reconstruct the likely timing, origins, and natural history of coinfecting strains to document within-host recombination and identify putative selection pressures. The approaches we have taken allow us to generate testable hypotheses about the factors governing HCMV evolution and pathogenesis in its natural host and are likely to be important to the rationale use, design, and impact of preventative and therapeutic interventions in the future.

## Methods

Approval for use of anonymized residual diagnostic specimens were obtained through the University College London/University College London Hospitals (UCL/UCLH) Pathogen Biobank National Research Ethics Service Committee London Fulham (Research Ethics Committee reference: 12/LO/1089). Informed patient consent was not required. Nucleic acid was enriched using custom baits and sequenced as previously described (30–36). All sequences were analyzed by the same methods (*SI Appendix*). The method used to calculate diversity is given (*SI Appendix*). The haplotypes were assembled using *HaROLD*, a newly developed maximum likelihood method (12) (*SI Appendix*).

Raw sequencing data for HCMV have been deposited in the European Nucleotide Archive under project accession no. PRJEB12814.

Raw data for VZV, EBV, HSV, HBV, HCV, HIV, Influenza, and Norovirus have been deposited as part of other studies.
